# The different meanings of tolerating the gut microbiome

**DOI:** 10.1128/mbio.01736-24

**Published:** 2026-03-03

**Authors:** Vinicius Mendes Vidal, Elena Montes-Cobos, Fábio B. Canto, Marcelo Torres Bozza

**Affiliations:** 1 Laboratório de Inflamação e Imunidade, Departamento de Imunologia, Instituto de Microbiologia Paulo de Góes, Universidade Federal do Rio de Janeiro 28125, Rio de Janeiro, Brazil; 2 Laboratório Multidisciplinar de Pesquisa, Departamento de Clínica Médica, Faculdade de Medicina, Hospital Universitário Clementino Fraga Filho, Universidade Federal do Rio de Janeiro 28125, Rio de Janeiro, Brazil; 3 Laboratório de Microbiologia e Imunologia IV, Departamento de Microbiologia, Imunologia e Parasitologia, Faculdade de Ciências Médicas, Universidade do Estado do Rio de Janeiro 28130, Rio de Janeiro, Brazil; 4 Laboratório de Tolerância Imunológica e Homeostase Linfocitária, Departamento de Imunobiologia, Instituto de Biologia, Universidade Federal Fluminense 28110, Niterói, Brazil; 5 Instituto de Investigação em Mucosas e Pele (INCT Mucosa e Pele), Belo Horizonte, Brazil; Instituto Carlos Chagas, Curitiba, Brazil

**Keywords:** tolerance, microbiome, gastrointestinal tract, Tregs

## Abstract

Multicellular life arose in a world dominated by microorganisms, a reality that has imposed a constant and pervasive selective pressure on all subsequent complex organisms. The immune system has been historically defined by its role in pathogen clearance through resistance mechanisms. However, a complementary and equally critical strategy is to enable the peaceful and inevitable coexistence with microorganisms, allowing each host species to shelter a unique associated microbiome. The term tolerance holds multiple meanings in immunology, yet all underlie a balanced and cooperative host-microorganism relationship. Each represents a different aspect of how the immune system limits tissue damage while maintaining functionality in the presence of microbial or inflammatory stimuli. Using the intestinal mucosa as a paradigm, we explore how epithelial barrier integrity, toxin neutralization, tissue repair, and stress response underpin disease tolerance; how microbial exposure calibrates innate immunity via epigenetic and metabolic reprogramming (LPS tolerance); and how the gut microenvironment fosters the generation of tolerogenic antigen-presenting cells and microbe-specific regulatory T cells to enforce immunological tolerance. We further explore how the microbiota itself is a potent inducer of these tolerogenic pathways and highlight IL-10 as a major hub, connecting different tolerogenic circuits. Finally, we examine the hygiene hypothesis, arguing that lifestyle changes during the Anthropocene disrupt these finely tuned tolerance mechanisms, thereby contributing to the rising incidence of immune-mediated diseases. We posit that these tolerance programs are fundamental prerequisites for engendering host-microbiota symbiosis, a relationship forged over millennia of co-evolution and endangered in the contemporary world.

## INTRODUCTION

**Fig 1:**
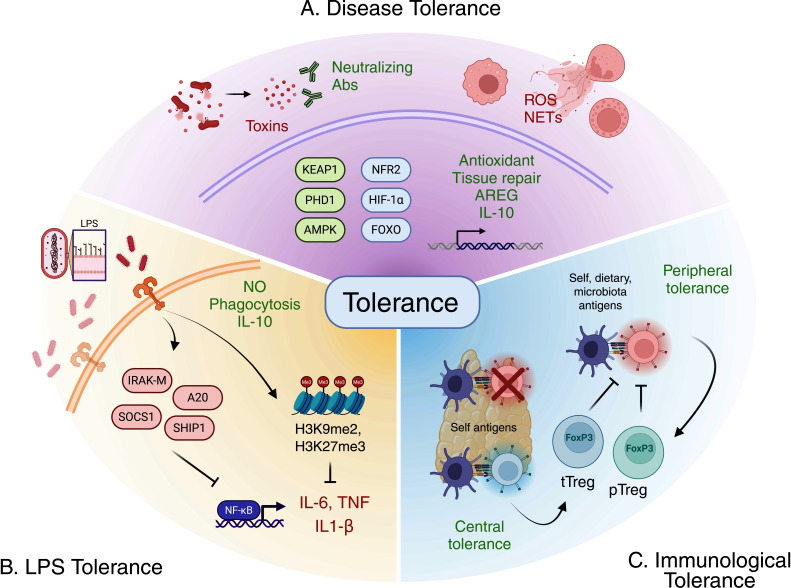
Three distinct perspectives of tolerance. (**A**) Disease tolerance limits the detrimental consequences of infection, whether directly caused by pathogen virulence factors or by the ensuing immune response. Its mechanisms include toxin neutralization, tissue repair, and the control of inflammation. Concurrently, intracellular sensors—such as KEAP1, PHD1, and AMPK—detect infection-induced metabolic perturbations like oxidative stress and nutrient deprivation. This detection activates stress-response pathways governed by the master regulators NRF2, HIF-1α, and FOXO, respectively. (**B**) Endotoxin tolerance, triggered by repetitive LPS exposure, diminishes inflammatory cytokines (e.g., IL-6, TNF, IL-1β) while maintaining effector functions like phagocytosis, nitric oxide (NO) release, and IL-10 production. This phenotype is reinforced epigenetically by repressive histone methylation on cytokine genes, and molecularly by the negative regulators IRAK-M, SOCS1, A20 (TNFAIP3), and SHIP1. (**C**) Immunological tolerance is an active process that safeguards against the improper activation of T cell clones. It targets those that are excessively reactive not only to self-antigens but also to innocuous non-self antigens encountered daily, such as dietary components or microbiota-derived molecules. This process is achieved through two main mechanisms: central tolerance, established in the primary lymphoid organs, prevents the export of highly self-reactive clones via clonal deletion. Meanwhile, peripheral tolerance acts in the tissues to suppress the activation of escaped self-reactive or environmentally reactive clones, a function carried out by regulatory T cells (Tregs) originating from the thymus (tTregs) or induced in the periphery (pTregs).

From their origin, multicellular organisms evolved under constant selective pressure from a microbial environment, a dynamic reflected in the deep evolutionary roots of the immune system (1). Even in basal lineages like Porifera and Cnidaria, organisms with a simple body plan and limited cellular differentiation, specialized cells that perform canonical immune functions are already present (2). The ancestral immunologic toolkit—composed of pattern recognition receptors (PRRs) (3, 4), antimicrobial molecules (5–7), cytokines (8), and epithelial barriers equipped with mucus-secreting cells (9, 10)—was established at the dawn of Metazoa evolution. Traditional immunology has largely been shaped by a defense-based worldview, casting the immune system in the role of a military force tasked with destroying microbial invaders. A formidable arsenal of resistance mechanisms, such as phagocytosis, reactive oxygen species (ROS), and antimicrobial peptides (AMPs), supports the mechanistic basis for this view. The clinical success of antimicrobial agents during infection further strengthens the perception that host health is fundamentally dependent on pathogen clearance (11). Beyond warfare, a second, cooperative function of the immune system is to negotiate a stable truce with the microbial world, which ultimately leads to host colonization by a unique microbiome. The microbiota, a consortium of bacteria, fungi, protozoa, and viruses, engages in persistent, dynamic, and symbiotic relationships with its multicellular host, forming long-term biological partnerships (12–16). Most of these host-microbiome interactions are mutually beneficial, a product of co-evolution (17, 18) that has rendered most (if not all) animal species dependent on their microbial partners for core physiological processes, including nutrition (19, 20), metabolism (21, 22), development (23, 24), behavior (15, 25, 26), and defense (27–30). The term tolerance holds multiple meanings in immunology, yet all underlie a biological diplomacy that limits tissue damage while maintaining functionality in the presence of microbial or inflammatory stimuli. Broadly, three conceptually distinct forms of tolerance can be recognized: disease tolerance, lipopolysaccharide (LPS) tolerance, and immunological tolerance (Fig. 1). Here we outline the conceptual foundations of these three types of tolerance and explore how they apply to the dynamic interplay between the host and its microbiota, with a particular focus on the intestinal mucosa as a paradigm of immune-microbial coadaptation.

## DISEASE TOLERANCE

Disease tolerance is a defensive strategy that limits the loss of host fitness during infection, mainly by restraining the damage caused directly by a microorganism (virulence) or indirectly by the immune and inflammatory response against it (immunopathology). It encompasses both the ability of cells, tissues, and organs to withstand damage and stress, as well as the reparative programs that restore structure and function. Most importantly, it preserves critical physiological processes decoupled from pathogen control, as it usually occurs independently of a reduction in microbial load (31–33). Since disease tolerance does not have a negative effect on pathogen fitness, it cannot fuel antagonistic coevolution (i.e., selection for counteradaptations in the microorganism) in the same way as is expected of resistance. Disease tolerance fulfills the theoretical evolutionary prerequisites required for forging host-microbe symbiosis and provides a framework for understanding our co-evolutionary history with the microbiota (34).

Heterogeneity in disease tolerance between individuals exposed to similar pathogen loads underlies the spectrum of clinical outcomes observed during infection. This is illustrated by the case of Mary Mallon (1869–1938), or “Typhoid Mary,” an asymptomatic carrier of *Salmonella enterica* serovar Typhi, that inadvertently caused outbreaks of typhoid fever (35). The recent COVID-19 pandemic evidenced profound inter-individual disease manifestations during SARS-CoV-2 infection (36), and how vaccines can protect the host from symptoms, but not from infection—thereby acting as promoters of disease tolerance.

The earliest characterization of disease tolerance was made in plants, based on the observation that two oat varieties (*Avena sativa*, Poaceae), Benton and Clinton 59, could differentially tolerate infection with the fungus *Puccinia recondita*. Despite equivalent fungal loads, the Clinton 59 cultivar suffered increased structural damage and yield reduction compared to the Benton variant (37). Disease tolerance is strongly influenced by genetic determinants. Humans with α^+^-thalassemia, a monogenic hemoglobinopathy, experience comparable *Plasmodium falciparum* parasitemia yet demonstrate reduced malaria-associated anemia and mortality (38). Mice expressing sickle hemoglobin survive experimental cerebral malaria. This protection occurs independently of parasite burden and requires heme oxygenase-1 (HO-1), indicating that sickle hemoglobin promotes host tolerance to *Plasmodium* infection (39). Similarly, inbred mouse strains display distinct tolerance capacities during *Plasmodium chabaudi* infection (40), and a systematic multi-strain murine screen recently revealed Rho GDP-dissociation inhibitor alpha (RhoGDIα or Arhgdia) as a negative regulator of disease tolerance during influenza virus infection (41). Primarily known for its role in kidney physiology (42), where loss-of-function mutations cause nephrotic syndrome (43, 44), Arhgdia orchestrates diverse cellular processes, including signaling (45), cytoskeletal dynamics, proliferation, survival (46), and insulin sensitivity (47). Although deletion of *Arhgdia* in epithelial cells reduces cell death during influenza challenge (41), the precise molecular mechanisms, cell-type-specific functions, and infection-context dependencies of Arhgdia-mediated tolerance remain elusive.

Environmental factors further recalibrate disease tolerance, dynamically shaping the set points established by genetics. For example, *Wolbachia* colonization protects *Drosophila melanogaster* from infection with Flock House Virus without altering viral load (48), whereas influenza virus infection compromises tolerance to secondary *Legionella pneumophila* challenge in mice (49). Xenobiotics can similarly shape tolerance states: doxycycline protects mice from bacterial sepsis without reducing pathogen load, instead acting through a mild and transient disruption of mitochondrial protein synthesis and electron transport chain activity (50).

The multicellular host deals constantly with the damage caused by microorganisms (51). Some microbes secrete toxins or release metabolic by-products that are noxious and even lethal at low doses to the host. Cell invasion and hijacking of intracellular machinery by pathogens often leads to cell lysis. Nutrient deprivation stemming from competition for critical resources such as iron and glucose may also prompt cell death (52, 53). Beyond direct microbial damage, host resistance mechanisms—including ROS, cellular cytotoxicity, and neutrophil-derived enzymes and DNA extracellular traps (NETs)—frequently cause collateral tissue injury (54). In several infectious diseases, the ensuing inflammatory response may contribute as much to pathology as does pathogen toxicity (55).

Tolerating the damage inflicted by microbial colonization is fundamental for host survival, but also for enduring the microbiota and eventually exploiting it. Mechanisms of disease tolerance encompass neutralization of toxins, tissue repair, inflammation control, metabolic adaptations, and cellular stress responses (39, 56, 57). Specific molecular sensors, including kelch-like ECH-associated protein 1 (KEAP1), prolyl hydroxylase domain protein 2 (PHD2), 5′ adenosine monophosphate-activated protein kinase (AMPK), nucleotide-binding domain leucine-rich repeat and pyrin domain-containing protein 3 (NLRP3), and heat shock proteins (HSPs), constantly monitor physiological parameters such as temperature, oxygen, osmolarity, and metabolite concentrations. Deviations beyond a critical threshold initiate defined stress responses—oxidative, hypoxic, osmotic, and metabolic—orchestrated by master transcriptional regulators like nuclear factor erythroid 2-related factor 2 (NRF2), hypoxia-inducible factor (HIF), forkhead box O (FOXO), nuclear factor of activated T cells 5, and heat shock factor protein 1 (HSF1). These factors drive metabolic adaptations in host cells to preserve essential cellular and tissue functions. Failure in this process frequently results in programmed cell death (31).

For instance, during *Plasmodium* infection, hemolysis releases free heme, inducing an oxidative stress response via KEAP1-NRF2-HO-1 axis. HO-1 activity clears heme but releases iron, which is cytotoxic at high intracellular concentrations. The iron-sequestering protein ferritin H chain (FtH) mitigates this cytotoxicity by storing excessive iron, a hepatoprotective mechanism critical in malaria. Increasing FtH function protects *Plasmodium*-infected mice without affecting parasite load—demonstrating that FtH metabolic response constitutes a disease tolerance mechanism (58). Infection with *Mycobacterium marinum* triggers protein kinase B (Akt)-FOXO signaling dysregulation in *D. melanogaster*, resulting in metabolic dysfunction and wasting syndrome. FOXO-mutant flies exhibit delayed mortality compared to wild-type controls without altering bacterial burden (59). Accordingly, upon either intestinal infection with *Salmonella* or lung infection with *Burkholderia thailandensis*, commensal strain *E. coli* O21:H+ translocates from the gut to white adipose tissue of mice. There, it triggers NLRC4/IL-18-dependent release of insulin-like growth factor 1 (IGF-1). Systemic IGF-1 subsequently activates phosphoinositide 3-kinase-Akt pathway in skeletal muscle, preventing muscle wasting and lethal disease (60). This highlights how a single commensal species can hamper infection-driven metabolic pathology (cachexia) and mortality by enforcing tolerance (60). Alterations in stress responses and metabolic pathways critically impact disease tolerance and survival trajectories during infection.

It has long been recognized that short-term exposure to low-level stressors can induce transient cellular adaptations that confer protection against subsequent challenges from either the same or unrelated stressors—a phenomenon termed hormesis (61). Heat and cold exposure, starvation, oxidative and hypoxic stress have been associated with lifespan extension and increased stress resilience in *D. melanogaster* (62–65), *Caenorhabditis elegans* (66–68), and mice (69–72). Notably, mitochondrial stress induced by xenobiotics such as tetracyclines can trigger mitohormesis, which protects mice from lethal bacterial sepsis (50) and influenza virus infection (73) without reducing pathogen burden, thereby promoting disease tolerance. Early-life stressors can also exert long-lasting effects on adult physiology (74), enhancing the organism’s ability to withstand stress- or pathogen-induced tissue damage (75). For example, exposure to cold, but not heat or hypergravity, improves *D. melanogaster* survival upon *Beauveria bassiana* fungal infection (76). While prolonged heat shock is deleterious, brief exposure enhances stress resilience and extends lifespan of *C. elegans* (77). This heat hormesis response is dependent on the HSP-HSF1 axis (78, 79) and might explain the extended healthspan observed in humans that regularly visit saunas (80). Some hormetic responses are dependent on microbiome changes: larval exposure to low-dose oxidants increases fruit-fly longevity through depletion of *Acetobacter* spp. from the gut microbiota, which otherwise promote age-associated gut dysfunction (81). Importantly, the nature, duration, and intensity of stressors determine whether outcomes are beneficial or detrimental (82–84), underscoring the complexity of hormetic processes. Therapeutic strategies targeting hormetic pathways and disease tolerance mechanisms represent promising approaches for preventing and treating infectious diseases.

## LPS TOLERANCE

Innate immune cells in the gut, particularly long-lived macrophages and dendritic cells (DCs), important antigen-presenting cells (APCs), are continuously exposed to microbial molecules from both commensals and pathogens, leading to profound adaptations that sustain homeostasis but can also drive inflammation and tissue damage. The gut microbiota represents a continuous, low-intensity immunological stimulus. Through the release of microbial-associated molecular patterns (MAMPs) such as LPS, peptidoglycan, lipoteichoic acids (LTA), flagellin, and microbial metabolites (short-chain fatty acids [SCFAs], bile acid [BA] derivatives, tryptophan catabolites), it continuously educates and calibrates innate immune cells in mucosal and systemic compartments. This constant dialog drives a continuum of innate immune states depending on signal intensity, duration, and metabolic context. The study of how the innate immune system modulates its responsiveness to repeated stimulation has evolved through nearly a century of experimental observations, leading to the recognition of three interrelated phenomena: the Shwartzman reaction, LPS (endotoxin) tolerance, and trained innate immunity. Although they represent distinct outcomes, these processes share a common theme: the capacity of innate immune cells to retain a functional memory of prior activation.

The first description of innate immune memory-like behavior dates back to 1928, when Shwartzman reported that two sequential injections of bacterial filtrates in rabbits produced localized hemorrhagic necrosis at the site of the first inoculation (85). These observations revealed that prior exposure to bacterial components could amplify inflammatory responses, suggesting an early form of innate immune sensitization. Later studies identified macrophages, neutrophils, and endothelial cells as key players, with tumor necrosis factor (TNF), IL-1, and coagulation factors mediating the pathological amplification of inflammation (86–88). The Shwartzman reaction thus represents the hyperinflammatory extreme of innate reactivity—an early paradigm for what would now be considered pathological innate immune training.

In contrast, Beeson (89, 90) described the opposite phenomenon, termed “tolerance to bacterial pyrogens.” Rabbits repeatedly injected with endotoxin became refractory to further febrile responses. Subsequent work demonstrated that this “endotoxin tolerance” reflected a state of reduced systemic inflammation, in which macrophages and monocytes produced markedly fewer inflammatory cytokines upon restimulation. In fact, it was demonstrated that LPS tolerance was cell-intrinsic, occurring primarily in macrophages that had been exposed to LPS *in vivo* or *in vitro* (91–93). The identification of toll-like receptors (TLRs), as critical PRRs recognizing MAMPs, and TLR4 as the LPS receptor enabled the dissection of signaling pathways underlying tolerance (94, 95).

LPS tolerance is a phenomenon in which macrophages, monocytes, and DCs acquire an adapted state following an initial exposure to LPS. The magnitude and duration of TLR-induced inflammation are tightly controlled by multiple regulatory mechanisms, including the induction of inhibitory molecules that dampen TLR signaling, the secretion of anti-inflammatory cytokines, and context-dependent remodeling of the TLR signaling complex.

Tolerant cells display attenuated production of pro-inflammatory cytokines such as TNF, IL-1β, and IL-6, accompanied by reduced expression of costimulatory molecules and suppressed activation of the myeloid differentiation primary response 88 (MyD88)-nuclear factor kappa B (NF-κB) pathway. This reprogramming is reinforced by the induction of negative regulators including interleukin-1 receptor-associated kinase M (IRAK-M), suppressor of cytokine signaling 1 (SOCS1), tumor necrosis factor alpha-induced protein 3 (TNFAIP3, or A20), and src homology 2 domain-containing inositol polyphosphate 5-phosphatase 1 (SHIP1), which collectively modulate both NF-κB and mitogen-activated protein kinase (MAPK) signaling cascades, establishing a state of controlled, anti-inflammatory responsiveness (96). Despite this suppressed inflammatory response that contributes to reduced tissue damage, cells often retain or even enhance their antimicrobial and anti-inflammatory functions, such as increased phagocytosis, nitric oxide production, and IL-10 secretion (97, 98).

Epigenetic analyses revealed that tolerance is driven by gene-specific chromatin remodeling, with pro-inflammatory genes becoming silenced through repressive histone marks (H3K9me2, H3K27me3), whereas antimicrobial and tissue-repair genes remain accessible (98). This selective transcriptional silencing explains how tolerant macrophages maintain key defense and regulatory functions while suppressing potentially harmful inflammation.

Similar to LPS tolerance, trained immunity involves the epigenetic reprogramming of innate immune cells. However, unlike tolerance, which limits inflammation, trained immunity enhances cytokine production (e.g., TNF, IL-6, IL-1β) and antimicrobial activity upon secondary challenge, even against heterologous pathogens. The phenomenon was described in the context of Bacillus Calmette-Guérin (BCG) vaccination and fungal β-glucan exposure, where prior stimulation conferred heightened protection against unrelated infections (99, 100). Mechanistically, trained immunity also depends on epigenetic priming, marked by activating histone modifications such as H3K4me3 and H3K27ac, and on metabolic rewiring toward aerobic glycolysis and glutaminolysis, governed by the mechanistic target of rapamycin (mTOR)–HIF-1α axis (101).

The concept that trained immunity is encoded at the level of hematopoietic progenitor cells was first established in murine models and later confirmed in humans (102–104). These studies demonstrated that innate immune memory can be imprinted within the hematopoietic compartment, leading to the long-term generation of myeloid cells with enhanced functional and metabolic properties.

In the gut, where tissue macrophages are continuously replenished by monocyte-derived cells (105, 106), such long-term reprogramming of hematopoietic progenitors likely contributes to the physiological state and functional set point of newly recruited macrophages. This hematopoietic imprinting thus provides a mechanistic bridge between innate and adaptive immunity, with broad implications for mucosal homeostasis, vaccine responses, host defense, and chronic inflammatory diseases.

## IMMUNOLOGICAL TOLERANCE

Immunity—classically defined as the long-term state of resistance to infections, tumors, or tissue transplants driven by the immune system—was long considered the default outcome of the immune system’s encounter with non-self and/or altered self molecules. Original observations by Ray Owen (107), however, challenged this paradigm and provided the pioneering description of immunological tolerance, a biological status contrary to immunity, as it warrants preservation, as opposed to elimination/exclusion, of the antigenic source.

Owen reported that adult dizygotic cattle twins, which had exchanged blood as embryos (108), maintained lifelong chimerism, harboring erythrocytes from their genetically dissimilar sibling (107–109). This revealed that the immune system was not inherently skewed to provide immunity against non-self molecules. In parallel, Medawar and colleagues showed such twins did not reject each other’s skin grafts (110). Using inbred mouse strains, they demonstrated that exposure to allogeneic cells during embryonic or neonatal windows, but not later in life, led to lifelong acceptance of donor-matched skin grafts in immunocompetent adult recipients (111–116)—designated actively acquired tolerance (111).

The experimental demonstration of immunological tolerance directly influenced Burnet’s Clonal Selection Theory (117), which interpreted the prevention of autoimmunity as a consequence of the destruction of lymphocytes bearing high-affinity receptors for self-ligands during early stages of development. Therefore, from this perspective, immunological tolerance relied on the lack of lymphocyte reactivity toward specific antigens, a notion that prevailed for decades in the field.

Besides providing a creative explanation for the avoidance of autoimmunity despite the inevitable emergence of self-reactive clones, Burnet’s theory proposed that the individual’s self was not exclusively determined by the genome but, otherwise, would be dynamically shaped by the environmental exposure to varied antigenic molecules during development. The “self-landscape” comprised those antigens (regardless of being encoded by the individual’s genome or derived from extrinsic sources) present in the body within the embryonic and/or perinatal time windows (117). The antigenic repertoire challenging the animal during those narrow ontogenetic stages would not trigger immunity but, instead, tolerance.

Mechanistic studies later suggested that thymus-derived lymphocytes were not only mediators of immunity but also indispensable for the establishment of immunological tolerance (118–128). The thymus exports, from the 3rd day after birth, specialized CD4^+^ T cells endowed with autoimmune-suppressive properties, in turn required to avoid the post-thymic activation of self-reactive clones (129–131). Therefore, the peripheral compartment would be seeded with a heterogeneous pool of CD4^+^ T lymphocytes, comprising ones with pathogenic potential and others with suppressive abilities.

After frustrated attempts to phenotypically characterize suppressor T lymphocytes, different studies independently confirmed that the peripheral CD4^+^ T cell pool could indeed be split, on the basis of surface cell markers, into both suppressive and pathogenic subsets that peacefully coexist in the post-thymic environment of a normal animal (132–143). Through adoptive transfers of purified CD4^+^ T cell subsets into T cell-deficient rodents, the suppressive subset (CD5^hi^/CD25^hi^ or CD45RB^lo^) was shown to be indispensable to prevent autoimmune reactions and microbiota-dependent inflammatory bowel disease (IBD) elicited by CD5^lo^/CD25^lo^/CD45RB^hi^ cells (131, 133, 137–141). Importantly, these findings confirmed that (i) intra-thymic negative selection (144–149) does not eliminate all potentially pathogenic T cell clones even under the presentation of autoimmune regulator (Aire)- and forebrain-expressed zinc finger 2 (Fezf2)-driven tissue-restricted antigens to developing thymocytes (150–152) and (ii) clonal anergy (153–155) is insufficient to prevent the peripheral activation of those hyperreactive lymphocytes (156).

The role of suppressor T lymphocytes—later designated regulatory T cells (T_REG_)—as mediators of peripheral tolerance was consolidated when the molecular driver of the suppressive program, the transcription factor forkhead box P3 (Foxp3), was discovered. Mutations in the murine *Foxp3* gene, and in its human ortholog *FOXP3*, were initially implicated as the cause of X-linked lethal autoimmune syndromes in both species (157–160). Due to the similarities between such spontaneous lymphoproliferative manifestations and those experimentally induced under T_REG_ deprivation, Foxp3 was investigated as an inducer of the T cell-mediated suppressive activity. In fact, a few years later, three groups simultaneously reported that Foxp3 is absolutely required for both T_REG_ cell development and function (161–163).

Ever since, the scope of physiological actions exerted by T_REG_ cells expanded beyond the prevention of immunopathology to the mediation of tissue repair (164, 165) and the promotion of a balanced relationship between the host and innocuous molecular sources, such as commensal microbiota, dietary macromolecules, and fetal alloantigens (166–186). Importantly, Foxp3^+^ T_REG_ cell subsets exported from the thymus (tT_REG_) or differentiated from CD4^+^Foxp3^-^ T lymphocytes in the peripheral compartment (pT_REG_) share a common transcriptional program but harbor distinct TCR reactivities, being cooperatively required for the prevention of autoimmunity and IBD (166, 167). Conversely, the establishment of allotolerance toward the fetus and tissue grafts (168–170), induction of oral tolerance to food antigens (171, 172), and maintenance of a diverse community of microbial symbionts at the mucosal surfaces (173, 174) rely on the actions exerted by pT_REG_ cells, which are physiologically induced under the supportive environment of the gut mucosa through microbiota- and dietary-derived signals (175–182). Germ-free mice indeed harbor diminished numbers of pT_REG_ cells, especially in the colon (178, 179), which is colonized by the most abundant and diversified community of microorganisms in the body. Reciprocally, pT_REG_ cells shape microbial composition in the intestinal lumen (173, 174, 183). In support of these findings, disturbances in the microbiota-pT_REG_ relationship lead to food allergy and IBD (184–186).

For many years, immune tolerance has been interpreted as a recessive phenomenon whereby T and B cell clones harboring high-affinity receptors toward self-antigens would undergo either physical elimination—that is, clonal deletion—within their site of development or permanent functional inactivation (clonal anergy) as mature lymphocytes in the periphery. Therefore, immunologic tolerance would be established as a biological state characterized by the lack of immune reactivity toward peripheral antigens under the steady state. The discovery of T_REG_ cells has revolutionized this paradigm, allowing a new conceptualization of immunological tolerance. In a contemporary sense, this phenomenon may be defined as a physiological process that prevents deleterious immune reactions to tissues through the inhibition—both as a consequence of recessive and dominant mechanisms operating in central and peripheral lymphoid organs—of adaptive immunity toward self- and non-self antigens.

## TOLERATING THE GUT MICROBIOME

**Fig 2:**
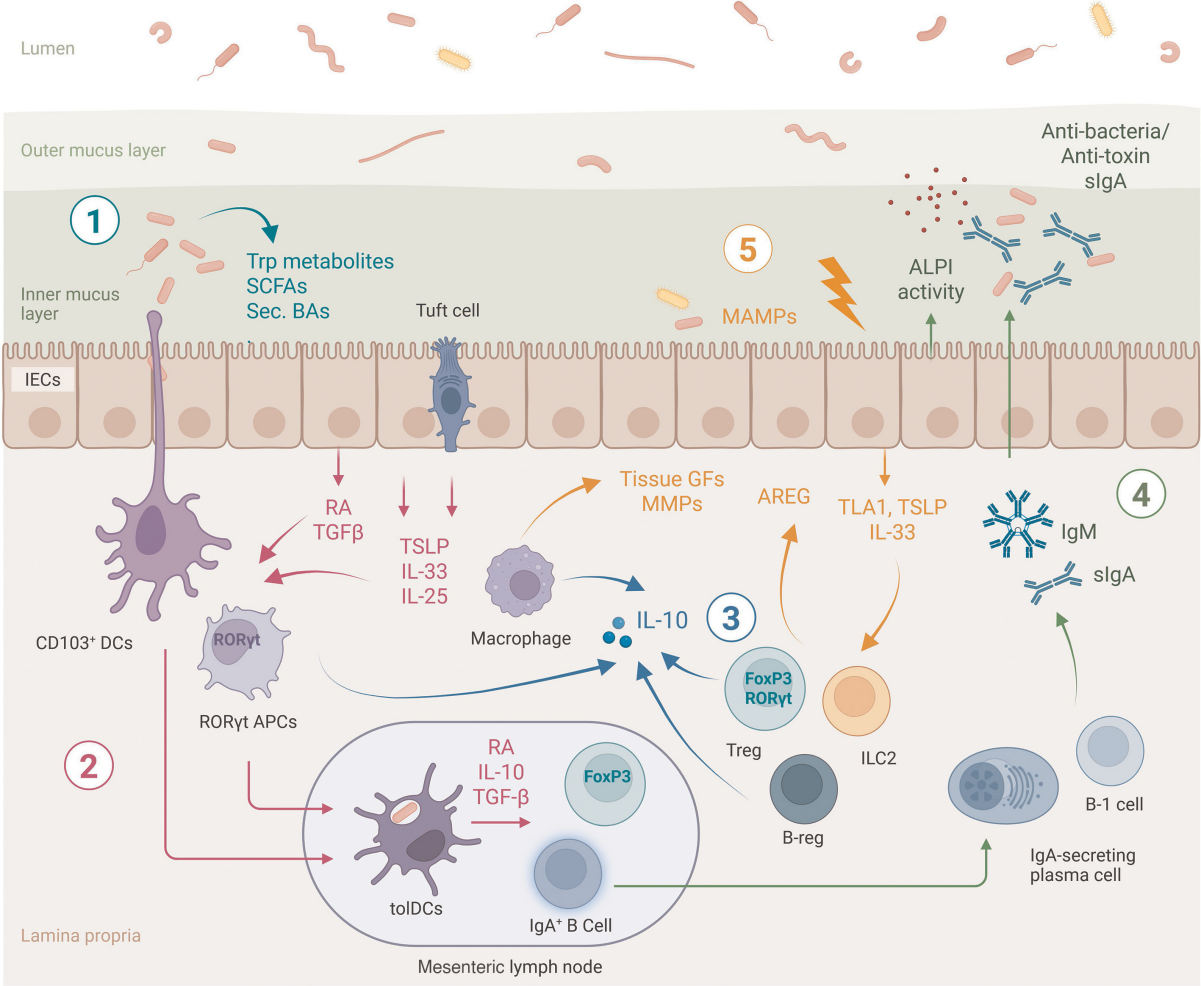
Mechanisms involved in tolerance of the gut microbiota. The gut microbiota promotes immune tolerance through several interconnected mechanisms (1). Firstly, microbial byproducts, such as SCFAs, tryptophan metabolites, and secondary BAs, constitutively reinforce the epithelial barrier (2). This barrier is further supported by tolerogenic immune responses: epithelial-derived factors like retinoic acid (RA), TGF-β, TSLP, IL-33, and IL-25 induce tolerogenic dendritic cells (tolDCs), which in turn support the generation of microbiota-specific regulatory T cells (Tregs), B cells (Bregs), and IgA-producing plasma cells (3). A cornerstone of this tolerogenic environment is the cytokine IL-10, produced by diverse mucosal immune cells, which is essential for maintaining homeostasis (4). Furthermore, secretory IgA (sIgA) and IgM antibodies control microbial populations and neutralize toxins, a function complemented by enzymatic mechanisms like intestinal alkaline phosphatase (ALPI) (5). Finally, active tissue repair mechanisms preserve barrier integrity. Upon microbial or damage signals, epithelial factors such as TL1A, TSLP, and IL-33 stimulate amphiregulin (AREG) expression by ILC2s and Tregs, while lamina propria macrophages enhance tissue remodeling through the release of growth factors and matrix metalloproteases (MMPs).

Multicellular hosts often harbor microbial communities at barrier surfaces—specialized tissues that provide protection from external threats while allowing selective molecular exchange. Mucosal compartments exhibit inherent permeability that enables essential processes, such as digestion by the gastrointestinal tract. Each host species shelters a distinctive microbiome, which exhibits considerable inter-individual variation (187–189), even among inbred lab mice created in different vivaria (190). Additionally, within a single host, microbial composition differs significantly across anatomical barriers (191), upon environmental changes (e.g., diet, xenobiotic exposure) (192, 193), and throughout development and life (194). Certain animals, such as bees, harbor a gut microbiota composed of a limited number of bacterial species (195), whereas humans shelter a vastly more complex consortium of trillions of bacteria belonging to thousands of species. The mammalian intestinal mucosa, therefore, constitutes a distinctive, intricate, and compelling environment for investigating how hosts achieve tolerance amidst an extraordinarily high load of diverse microbes. This homeostatic equilibrium is mediated by dynamic, tightly regulated, and non-redundant immune mechanisms operating in the gut (Fig. 2).

Microorganisms exhibit context-dependent behavior, which poses a challenge for their classification as commensals, pathobionts, or pathogens (196). For instance, *Escherichia coli* is a beneficial gut commensal of wild-type mice but becomes pathogenic in interleukin-2-deficient (*Il2^-/-^*) mice. Conversely, certain *E. coli* strains, such as enterohemorrhagic *E. coli*, are highly pathogenic even in immunocompetent hosts. Similarly*, Bacteroides vulgatus* is pathogenic in HLA-B27/β2m transgenic rats but protects *Il2^-/-^* mice from disease mediated by wild-type *E. coli* (197, 198). Traditionally, a pathobiont is a symbiont capable of inducing pathology only under specific genetic or environmental perturbations in the host (196). Current definitions often overlook the intra-specific genetic diversity of both hosts and microorganisms and require careful contextual application (199).

### Epithelial barrier, tissue damage, and repair

The architecture of the intestinal mucosa is broadly defined by a single layer of mucus-producing epithelial cells and an underlying loose connective tissue, the lamina propria. Gut microorganisms inhabit distinct niches within the mucus layer (200), and their interactions with host cells require precise regulation to prevent overt microbial translocation or excessive inflammatory stimulation. This balance is maintained by dynamic and tightly coordinated processes collectively termed barrier function, which encompass mucus production (201, 202), epithelial intercellular adhesion proteins, AMP secretion (203–205), epithelial turnover and repair, and protein fucosylation and glycosylation (206). Failure in barrier function and subsequent breach can result in pathological inflammation, dysregulated fluid balance, and nutrient uptake (207, 208). Therefore, mechanisms that repair the epithelium and foster barrier integrity drive tolerance in the gut (34).

The tonic recognition of commensally derived MAMPs by epithelial and immune cells promotes barrier repair (209, 210). PRR activation by MAMPs is critical to restore homeostasis after chemically induced tissue damage with dextran sulfate sodium (DSS). Mice with impaired MAMP-TLR signaling either due to TLR2-, TLR4-, and MyD88-genetic deletion or extensive antibiotic use are more likely to die from DSS-induced colitis (211). Administration of TLR ligands LPS or LTA to antibiotic-treated mice is sufficient to revert disease susceptibility by promoting epithelial repair (211). Although excessive inflammation is deleterious for gut homeostasis, activation of the inflammasome and IL-18 production are critical for the maintenance of barrier function (212–214).

Tissue repair is coordinately driven by several immune and stromal cells that are responsible for inducing cellular proliferation, recruitment, and differentiation. This process requires the precise deposition of extracellular matrix components and subsequent remodeling, whose failure leads to fibrosis, scarring, and tissue dysfunction (215). The competence of this entire system defines a tissue’s inherent tolerance to damage, creating a stark physiological gradient between highly regenerative sites like the intestinal mucosa and low-tolerant compartments like the central nervous system (32). In the gut, a major regulator of barrier function and repair is IL-22 (216). For instance, IL-22 promotes disease tolerance during *Citrobacter rodentium* infection through fucosyltransferase 2 (Fut2)-dependent α(1,2)-fucosylation of intestinal epithelial cells (IECs) (217). Additionally, upon damage and stress, IECs release alarmins IL-33, TNF-like ligand 1A (TL1A), and thymic stromal lymphopoietin (TSLP), which can lead to both inflammatory and repairing responses (218–221). IL-33-ST2 signaling is particularly important for polarizing Foxp3^+^ T_REG_ cells and type 2 innate lymphoid cells (ILC2s) toward a repairing program marked by amphiregulin (AREG) expression. AREG, alongside other epithelial growth factors, is induced in response to injury and is key for tissue regeneration (222–224).

Neutralizing microbially derived exo- and endotoxins is also necessary to tolerate the microbiome. LPS is constitutively detoxified by dephosphorylation of the lipid A moiety through intestinal alkaline phosphatases (ALPI) expressed by IECs (225). ALPI expression is constitutively induced by the microbiome and crucial to prevent intestinal inflammation, since ALPI-deficient zebrafish develop spontaneous gut inflammation unless raised in germ-free conditions (225). ALPI can be further induced by resolutive lipid mediator Resolvin-E1, protecting mice from DSS-induced colitis (226). In mammals, mucosal antibodies, mainly secretory IgA (sIgA) and IgM, are additional tools for neutralizing toxins secreted by enteropathogens like *Vibrio cholerae*, *Shigella flexneri*, and *C. difficile* (227). Since toxin neutralization does not affect bacteria directly, it protects the host by limiting tissue damage. Beyond neutralization, sIgA is also employed by the host as a tool for selecting commensals. Colonization of germ-free mice with *Bacteroides fragilis*—a key inducer of regulation in the gut—is impaired in IgA-deficient animals (228). In addition, Foxp3^+^ T_REG_ cells promote the diversification of IgA repertoire in the Peyer’s patches, thereby favoring the selection of a microbial community with reciprocal IgA- and T_REG_-supportive properties (183).

Microbiome tolerance in invertebrates reveals fundamental mechanisms that operate autonomously from adaptive immunity, hence not involved with immunological tolerance. These animals pose interesting frameworks to disentangle tolerance mechanisms. Genetic screens in *D. melanogaster* have identified mutants with divergent survival outcomes to *Listeria monocytogenes* infection despite equivalent microbial load (229). Notably, a mutation in the gustatory receptor gr28b induces anorexic behavior in fruit flies that enhances disease tolerance to *Salmonella* infection, yet simultaneously impairs resistance to *Listeria monocytogenes* (230), demonstrating that a single genetic mutation can have opposing, pathogen-specific effects on resistance and tolerance pathways.

### Microorganisms induce tolerance

Microbiota enforces host tolerance through the secretion of many metabolites, which is also dynamically shaped by host diet and xenobiotic exposure (231, 232). SCFAs fermented from soluble dietary fiber serve as the primary energy source for IECs, promote inflammasome-tonic activation (233), exert potent immunoregulatory effects, and protect from enteropathogenic infection (207, 234, 235). They polarize many immune cells toward a tolerogenic phenotype by engaging G protein-coupled receptors GPR41/GPR43 (236, 237) and inhibiting histone deacetylases (HDACs) (238). Notably, butyrate is a key driver of Foxp3^+^ pT_REG_ generation and function (239–241). In the colon, microbiota-converted secondary BAs alongside SCFAs foster the generation of protective retinoic acid-related orphan receptor gamma t (RORγt)^+^Foxp3^+^ T_REG_ cells (180, 242, 243), which mediate the local suppression of exacerbated inflammation (180, 181), a balanced relationship with the microbiome (184, 244), and the extra-enteric tissue repair (245). Furthermore, microbiota-derived tryptophan catabolites signal through aryl hydrocarbon receptor (AhR) to reinforce mucosal tolerance. AhR activation orchestrates a multi-faceted program that drives xenobiotic detoxification response (246), reinforces barrier integrity (247–250), and supports generation/function of several cells, including Foxp3^+^ T_REG_ cells (251, 252), Foxp3^-^ Tr1 cells (246, 252, 253), Tγδ intraepithelial lymphocytes (IELs) (254), T_H_17 cells (251, 255), and ILCs (256). Indeed, AhR functions as a molecular integrator of the indoleamine-2,3-dioxygenase-1 (IDO1) pathway during LPS tolerance, which protects mice against immunopathology from *Salmonella* infection (257), thereby linking innate reprogramming directly to disease tolerance.

Early-life host-microbiome interactions, starting with the colonization by foundational species, program fundamental aspects of mammalian development and immune education with long-lasting consequences for the host (258–260). The postnatal establishment of the microbiota is a prerequisite for the development of mucosal-associated invariant T (MAIT) cells. Microbiota-derived riboflavin translocates to the thymus and enables major histocompatibility complex class I-related gene protein (MR1)-dependent generation of MAIT cells. Following their thymic education, MAIT cells home to barrier sites and are poised to orchestrate epithelial repair post-injury (261). Breastfeeding for the appropriate period is also essential for the proper maturation of the immune system. A specific immunological window during the transition from milk to solid food, referred to as the weaning reaction, allows for the expansion and shift of the microbiota. This microbial transition, in turn, induces the generation of RORγt^+^Foxp3^+^ T_REG_ cells. Premature weaning, or weaning in the context of antibiotic therapy, abrogates the development of this T_REG_ subset, thereby imprinting a lasting susceptibility to exacerbated immune-mediated diseases in adulthood (244).

Host-microbiome symbiosis is a bidirectional process, wherein commensals actively engage host pathways to foster the very tolerogenic environment that ensures their persistence. For instance, polysaccharide A (PSA) from the commensal symbiont *B. fragilis* is a potent inducer of Foxp3^+^IL-10^+^ pT_REG_ cells in a TLR2-dependent manner (262). The presence of *B. fragilis* is beneficial to the host, being sufficient to protect against the development of various inflammatory pathologies (263). The colonization of germ-free mice with the minimally diverse microbial consortium Altered Schaedler Flora (ASF) drives the expansion and differentiation of colonic Foxp3^+^ pT_REG_ cells in a MyD88/TRIF-dependent process (179). Conditional MyD88 deficiency in Foxp3^+^ cells leads to reduced T_REG_ cell number and function in the lamina propria of small and large intestines, as well as in the Peyer’s patches; as a result, mice develop disturbed IgA response, altered microbial colonization, and enhanced susceptibility to gut inflammation (264). Notice that the same innate microbial recognition mechanisms, typically associated with inflammatory cascades, are co-opted in the mucosa to enact tolerogenic programs instead. Sometimes even parasites/pathogens can be inducers of tolerogenic circuitries, either because this confers selective advantage to the parasites that exploit host tolerance, and/or because it is the current best evolutionary solution for the host, a trade-off that limits immunopathology. The colonization with the nematode *Syphacia obvelata* also expands Foxp3^+^ T_REG_ cells and IL-10 production, which, while potentially aiding parasite persistence, also confers benefits to the host as it mitigates intestinal inflammation (265). Therefore, the microbiota is a physiological instructor of immunoregulation, actively shaping tolerance in the intestinal mucosa.

### Tolerogenic APCs and pT_REG_ cells

The gut is unique because APC differentiation occurs in constant contact with microbial and epithelial-derived signals, which sculpt their phenotype and function. In the intestinal mucosa, DCs continuously integrate cues from epithelial cells, stromal networks, and commensal microorganisms to maintain a delicate balance between tolerance and immunity. Among the diverse intestinal APC subsets, a specialized population of tolerogenic DCs plays a central role in preserving mucosal homeostasis and preventing excessive inflammation (266, 267). Several microenvironmental factors contribute to this tolerogenic imprinting. Epithelial-derived transforming growth factor-beta (TGF-β) and retinoic acid (RA) are key mediators that induce integrin αE (CD103) expression on developing DCs and endow them with the ability to produce RA, TGF-β, and IL-10, essential for the generation of Foxp3^+^ pT_REG_ cells. Stromal cells and epithelial tuft cells further shape this program through the secretion of TSLP, IL-33, and IL-25, which suppress pro-inflammatory cytokine production and favor the maintenance of a semimature DC phenotype. In parallel, commensal-derived MAMPs provide tonic stimulation through TLRs and NOD-like receptors, sustaining basal activation of DCs without triggering full inflammatory maturation. This low-grade PRR signaling induces NF-κB-dependent chromatin remodeling and epigenetic reprogramming that stabilize a hyporesponsive, IL-10-competent state reminiscent of innate immune tolerance. As evidence of the complex interactions between the microbiota and the gut immune system, TLR9/MyD88-dependent stimulation of small intestine DCs by bacterial CpG represses local pT_REG_ differentiation (268), thus controlling the number of such cell subset under the steady state.

Recent studies have identified specialized subsets of tolerogenic APCs in the intestinal lamina propria and mesenteric lymph nodes (mLNs) that play central roles in the induction of Foxp3^+^ pT_REG_ cells. Classical CD103^+^ DCs are phenotypically characterized by CD103^+^CD11b^+^ or CD103^+^CD11b⁻ profiles and functionally by their ability to promote T_REG_ differentiation, IgA class switching, and epithelial barrier maintenance. More recently, RORγt^+^ APCs have emerged as critical drivers of pT_REG_ generation in response to the microbiota (269–274). These include DCs, as well as the rarer Thetis cells (TCs) and Janus cells (JCs)—cellular types with mixed features of medullary thymic epithelial cells (mTECs) and ILC3s. Collectively referred to as RORγt^+^ APCs, these populations express MHC-II and directly induce pT_REG_ differentiation in the intestinal mucosa and draining lymph nodes. Deletion of MHC-II from all RORγt^+^ APCs profoundly impairs pT_REG_ induction in response to commensal bacteria and leads to spontaneous intestinal inflammation, underscoring their essential role in establishing peripheral tolerance to the microbiota (266, 267). Together, CD103^+^ DCs and RORγt^+^ APCs integrate microbial and epithelial cues to sustain mucosal homeostasis and ensure mutualistic coexistence between the host and its microbiota.

Thus, the intestinal microenvironment acts as a continuous instructor of DC differentiation, where epithelial factors, commensal MAMPs, and local metabolites synergize to generate a network of tolerogenic DCs. These cells limit PRR-driven inflammation, sustain epithelial integrity, and enforce immunological tolerance to the vast microbial community that inhabits the gut—a process whose failure underlies chronic inflammatory disorders such as IBD.

### IL-10 as a master regulator for the many types of tolerance in the intestinal mucosa

A foundational discovery in mucosal immunology came from the work of Kühn and colleagues, who demonstrated that IL-10 deficiency in mice triggers spontaneous colitis, thereby establishing a model for deciphering the cytokine’s immunoregulatory functions and its role in IBD pathogenesis (275). The translational significance of this pathway is underscored by the development of severe, early-onset IBD in humans with monogenic defects in the IL-10 signaling axis (276). Spontaneous colitis is completely dependent on the presence of commensal microorganisms, since germ-free *Il10^-/-^* mice remain disease-free (277). Importantly, exposure of *Il10^-/-^* mice to pathobionts under conventionally raising conditions (as opposed to specific pathogen-free) leads to earlier disease onset (275). Consistently, *Il10^-/-^* mice exhibit heightened susceptibility to develop gut inflammation when exposed to murine norovirus (278), *Helicobacter hepaticus* (279, 280), *C. rodentium*, *Bacteroides* spp. (281), and *Bilophila wadsworthia* (282). Following oral *Toxoplasma gondii* infection, *Il10^-/-^* mice have increased ileum inflammation and tissue damage, but similar parasitic load when compared to wild-type controls (283), indicating clearly that IL-10 promotes disease tolerance. Our group recently found that *Il10^-/-^* mice develop acute and lethal colitis when co-housed with macrophage migration inhibitory factor (MIF)-deficient mice. Importantly, *Il10^-/-^* mice survive when co-housed with wild-type mice from the same facility. *Mif^-/-^* mice shelter a unique gut microbiota composition, enriched in pathobionts/pathogens, yet they remain healthy—indicating that MIF impairs disease tolerance in the gut. Additionally, double-deficient *Mif^-/-^Il10^-/-^* mice develop spontaneous and very early onset IBD, indicating that IL-10 is necessary for disease tolerance in *Mif^-/-^* mice (284). Thus, IL-10 is a critical regulator of intestinal homeostasis, constraining inappropriate inflammatory responses and enabling gut microbiome colonization.

Similarly, mice with combined defects in IL-10 and TGF-β signaling (dnKO) develop severe, fully penetrant, and microbiota-dependent intestinal inflammation. This pathology is both preventable and reversible with antibiotics and can be specifically initiated by reintroducing single *Bacteroides* species (i.e., *B. thetaiotaomicron*, *B. vulgatus*, or *Bacteroides* sp. TP5) into healthy antibiotic-treated dnKO mice. While these bacteria colonize dnKO and heterozygous control mice to comparable numbers, they elicit disease specifically in hosts with impaired IL-10/TGF-β signaling (281). Mechanistically, outer membrane vesicles from *B. thetaiotaomicron*, equipped with sulfatases that enhance their penetration into the lamina propria, are taken up by gut macrophages. Lack of IL-10R signaling in these macrophages abrogates MAMP tolerance, leading to unrestrained inflammatory activation (285). Importantly, while commensal *E. coli* blooms during active spontaneous colitis in dnKO mice, its transfer fails to initiate disease (281), confirming that its expansion is a consequence—not a cause—of the inflammatory milieu. Thus, dysbiosis and the expansion of certain microbial groups during gut inflammation can be secondary to a breakdown in host tolerance mechanisms toward commensal species that are resident at the same microbial density. This model illustrates the intricate connection between MAMP tolerance and disease tolerance in the gut compartment.

In the healthy intestine, IL-10 is produced by multiple immune and stromal cells, forming an integrated regulatory network that maintains mucosal tolerance. Resident macrophages and DCs are primary innate sources of IL-10, providing continuous feedback inhibition in response to commensal-derived signals. IL-10 produced by APCs regulates homeostatic T cell responses to the microbiota by suppressing CD4^+^ T_H_1 and T_H_17 effector cells that drive colitis. Among adaptive populations, Foxp3^+^ T_REG_ cells, particularly the colonic RORγt^+^ subset, are key contributors to the IL-10-rich mucosal environment that limits macrophage activation and effector T cell function. Loss of IL-10 specifically in T_REG_ cells results in spontaneous colitis, underscoring their essential role in maintaining intestinal immune regulation (286). Particularly, tolerance to *H. hepaticus* relies on the c-MAF-driven IL-10 expression by microbe-specific pT_REG_ cells; T_REG_-intrinsic c-MAF deficiency leads to accumulation of *H. hepaticus*-reactive T_H_17 cells in the large intestine and consequent development of gut inflammation (185) as well as to dysbiosis characterized by the enrichment of T_H_17-inducing bacteria (174). Regulatory B cells, IgA^+^ plasma cells, and subsets of ILCs further reinforce this anti-inflammatory circuit.

Beyond its role in adaptive immunity, IL-10R signaling in innate immune cells is also critical for mucosal homeostasis (287). Disruption of this pathway impairs the differentiation and function of anti-inflammatory macrophages, diminishes IL-10 production, and disrupts crosstalk between innate and adaptive immune cells, leading to uncontrolled intestinal inflammation (288). Thus, IL-10R signaling in both T cells and innate immune cells is indispensable for maintaining immune tolerance. Together, these IL-10-producing and IL-10-responsive populations form a redundant regulatory network that limits PRR- and antigen-driven inflammation, preserves epithelial integrity, and promotes mutualistic coexistence with the intestinal microbiota.

## THE LOSS OF TOLERANCE IN THE ANTHROPOCENE

Multicellular organisms have co-evolved with diverse microbial communities, evolving adaptive mechanisms to tolerate their presence. Concurrently, microbes have functioned as inducers of immunoregulatory pathways. Modern lifestyles in urban and industrialized environments have made humans lose contact with many organisms—commensals, parasites, pathobionts, and pathogens—with which our species had established intricate co-evolutionary relationships essential for the proper education of the immune system (289–293). Widespread use of antimicrobials, anti-helminthics, and Western diets has further reduced the diversity and the numbers of colonizing microorganisms and macroparasites (187, 294–297). This rapid and profound ecological shift reveals an evolutionary mismatch, where newly assembled metaorganisms conflict with our evolved biology – contributing to the rise of inflammatory, allergic, and autoimmune diseases in contemporary societies (298–303).
